# Metagenomic Analysis of Garden Soil-Derived Microbial Consortia and Unveiling Their Metabolic Potential in Mitigating Toxic Hexavalent Chromium

**DOI:** 10.3390/life12122094

**Published:** 2022-12-13

**Authors:** Nidhi Singh, Veer Singh, Sachchida Nand Rai, Emanuel Vamanu, Mohan P. Singh

**Affiliations:** 1 Centre of Bioinformatics, University of Allahabad, Prayagraj 211002, India; 2 School of Biochemical Engineering, Indian Institute of Technology, Banaras Hindu University, Varanasi 221005, India; 3 Centre of Biotechnology, University of Allahabad, Prayagraj 211002, India; 4 Faculty of Biotechnology, University of Agricultural Sciences and Veterinary Medicine of Bucharest, 011464 Bucharest, Romania

**Keywords:** garden soil, metagenome, microbial communities, elemental analysis, hexavalent chromium

## Abstract

Soil microbial communities connect to the functional environment and play an important role in the biogeochemical cycle and waste degradation. The current study evaluated the distribution of the core microbial population of garden soil in the Varanasi region of Uttar Pradesh, India and their metabolic potential for mitigating toxic hexavalent chromium from wastewater. Metagenomes contain 0.2 million reads and 56.5% GC content. The metagenomic analysis provided insight into the relative abundance of soil microbial communities and revealed the domination of around 200 bacterial species belonging to different phyla and four archaeal phyla. The top 10 abundant genera in garden soil were *Gemmata*, *Planctomyces*, *Steroidobacter*, *Pirellula*, *Pedomicrobium*, *Rhodoplanes*, *Nitrospira Mycobacterium*, *Pseudonocardia*, and *Acinetobacter*. In this study, *Gemmata* was dominating bacterial genera. *Euryarchaeota*, *Parvarchaeota*, and *Crenarchaeota* archaeal species were present with low abundance in soil samples. X-ray photoelectric spectroscopy (XPS) analysis indicates the presence of carbon, nitrogen–oxygen, calcium, phosphorous, and silica in the soil. Soil-derived bacterial consortia showed high hexavalent chromium [Cr (VI)] removal efficiency (99.37%). The bacterial consortia isolated from garden soil had an important role in the hexavalent chromium bioremediation, and thus, this study could be beneficial for the design of a heavy-metal treatment system.

## 1. Introduction

Microorganisms are the most abundant organism in the environment and are present everywhere on the earth, including solid waste, water, the human gut and soil ecosystems inhabited by an enormous plethora of microbial communities [1]. One soil sample predictably contains over 10^30^ microorganisms and thousands of archaea and eukaryotes [2]. In soil systems, the physiochemical properties of soil are influenced by bacterial communities [3]. Soil microbial communities are also affected by soil texture. The bacterial proportion is most dominant in the soil microbial communities [4,5]. The most common bacterial genera in grasslands are *Acidobacteria*, *Actinobacteria*, and *Proteobacteria* [6]. There are several studies on soil metagenomics that have been conducted, but only a few research studies have explained the role of soil microbial diversity in waste management and land use [7]. Land use has also been observed indirectly affecting the microbial community structure due to changes in soil characteristics such as pH, temperature, and elemental composition [8]. Some other factors, such as pesticides, fertilizer, and rainfall, also affect the soil microbial diversity [9]. 

Soil microbial diversity is very complex, and its culture is challenging for researchers. According to microbial evolutionary studies, about 1% of the bacterial species found in the environment can be cultured in the lab [10,11]. Various types of microbial diversity present in the soil are uncharacterized [12]. The metagenome approach is used to explain the core microbiota of many fields such as crops, soil, gut microbiome, wastewater, etc. [13]. Metagenomics is a culture-independent method used for the characterization of microbial diversity in soil samples [14]. It recognizes the diversity of uncultured soil microbial species by analyzing the direct isolation of genetic materials (DNA) from given habitats [15,16]. Rawat and Joshi [1] reported that Next-Generation-Sequencing technology has emerged as a valuable tool for analyzing biodiversity in metagenomic samples and metabolic pathways. Recently, the next-generation technique has been extensively used for microbial diversity analysis to address environmental concerns [17,18]. Next-generation sequencing (NGS) technologies that mark conserved regions of evolutionary indicators to find operational taxonomic units (OTUs) extend the analysis of complex microbial communities [19]. These approaches distinguish viruses, bacteria, fungi, parasites, and archaea from previously undiscovered and uncultivable organisms [20]. Soil microbial communities involve the highest level of prokaryote organisms in the environment [21].

Soil microbes, especially garden-soil microbes, have lignocellulosic degradation properties [22]. Soil microbes also play important role in suppressing plant diseases such as Clubroot disease, Root-Rot Disease, and Chinese Clubroot [23,24,25]. In addition, heavy metal-tolerant microbes isolated from soil have good heavy metal removal properties. It has been described that soil microbes encourage plant growth and enhance the phytoremediation of heavy metal ions [26]. Heavy metals such as arsenic (As), cadmium (Cd), chromium (Cr), lead (Pb), and mercury (Hg) cause several harmful effects such as kidney damage, heart failure, liver damage, and cancer [27,28,29]. Hexavalent chromium (Cr (VI)) is a carcinogenic heavy metal ion, and it has 100 times more toxicity than Cr (III) [30]. Cr (VI) comes in the effluent of several industrial activities such as chromate plating, leather tanneries, steel, and paint [31]. Several heavy-metal removal methods include reverse osmosis, chemical reduction, and precipitation use for the removal of Cr (VI) from wastewater [32]. These described heavy metal removal methods showed several limitations such as the production of secondary pollutants and the inability to remove heavy metals at very low concentrations of heavy-metal ions in the water [27,33]. Microbial removal of Cr (VI) is considered an eco-friendly, inexpensive and effective method. Several studies have been conducted on the microbial elimination of Cr (VI) from contaminated sites [34,35,36,37]. 

This study aimed to investigate the microbial diversity of garden soil. The elemental composition of the soil sample was also investigated. Additionally, the Cr (VI) removal efficiency of garden soil-derived microbial consortia was also explored in this study.

## 2. Materials and Methods

### 2.1. Sample Collection and Elemental Characterization

One garden soil sample was collected from the Banaras Hindu University campus, Varanasi, India (latitude 25°19′18.0624″ N, longitude 82°59′14.2404″ E) in November 2020. The temperature of the soil sample was 21 °C at the time of sample collection. The soil sample was stored at 4 °C in the laboratory until further analysis. The elemental composition, including phosphorus (P), calcium (Ca), nitrogen (N), silica (Si), and oxygen (O) was investigated through X-ray photoelectric spectroscopy (XPS) (Thermo Fisher Scientific make, Waltham, MA, USA). The sample for XPS analysis was prepared by mixing a 3:1 ratio of potassium dichromate (KBr) (HiMedia make, Mumbai, India) and soil sample [30]. 

### 2.2. DNA Extraction and Metagenomic Sequencing

The total DNA was extracted from a 1 g garden soil sample using commercially presented kits such as Qiagen, Zymo Research, and Thermo Fisher [38,39]. NanoDrop and gel electrophoresis were used to assess the quality of the extracted DNA [40]. The quality of DNA was estimated at 260/280 using NanoDrop, and the reading value aimed to stay in the range of 1.8 to 2. The DNA was amplified using polymerase chain reaction (PCR) at standard PCR conditions [40]. Primers 13F (5′AGAGTTTGATGMTGGCTCAG3′) and 13R (5′ TTACCGCGGCMGCSGGCAC3′) were used in PCR. The second nested PCR was used for the amplification of the entire variable region (V3-V4 hypervariable region) of 16S rDNA. The primers V13F (5′AGAGTTTGATGMTGGCTCAG3′) and V13R (5′ TTACCGCGGCMGCSGGCAC3′) were used for the amplification of the entire variable region. Eight further cycles of PCR were performed with Illumina barcoded adapters to prepare the sequencing libraries. AMPure beads were used to purify the libraries, and a Qubit dsDNA High-Sensitivity assay kit was used to quantify them. Next-generation sequencing was performed using an Illumina MiSeq with a 2x300 PE v3 sequencing kit.

### 2.3. Bioinformatics Analysis

The quality check of raw data was carried out using FastQC, then trimming low-quality reads using TRIMGALORE [41,42]. The trimmed reads were then processed further using the Uclust tool, which produced sequences that were combined and grouped into operational taxonomic units (OTUs), removed chimeras, and merged paired-end reads with an average 97% sequence similarity. An open-reference OTU was chosen and used to search the GreenGenes reference database [43]. QIIME2 workflows achieve OTU abundance calculation and estimation correction [44].

### 2.4. Isolation of Soil-Derived Microbial Consortia and Cr (VI) Removal Using Microbial Consortia

The mixed bacterial species were isolated from garden soil. First, 1 g of soil sample was mixed in 100 mL of Luria Bertani (LB) broth and incubated at 37 °C for 24 h. Next, 1 mL grown mixed bacterial culture was inoculated in Luria Bertani (LB) broth containing 100 mg/L Cr (VI). The effect of Cr (VI) on microbial growth was investigated for 24 h. The Cr (VI) removal efficiency of microbial consortia was also evaluated. The mixed bacterial culture was incubated for 6 days at pH 7, 37 °C, and 180 rpm. The samples were collected at 24 h intervals for 6 days. The bacterial culture was centrifuged at 5000 rpm for 10 min for cell separation from broth media. The remaining Cr (VI) concentration in the liquid media was determined using 1, 5-Diphenylcarbazide. The elimination of Cr (VI) from the liquid medium was analyzed using Equation (1).

(1) $$\%\;\mathrm{Cr}\;(\mathrm{VI})\;\mathrm{removal}=\frac{C_{o}-C_{e}}{C_{o}}\times 100$$

where *C_o_* and *C_e_* are the initial and final Cr (VI) concentrations in mg/L. 

### 2.5. Statistical Analysis

All experiments in this research were performed in triplicate (*n* = 3) and graphs were constructed using the average experimental data value. The experimental errors (±standard deviation) were identified and plotted as error bars in graphs.

## 3. Result

### 3.1. Elemental Composition of Garden Soil

**Figure 1:**
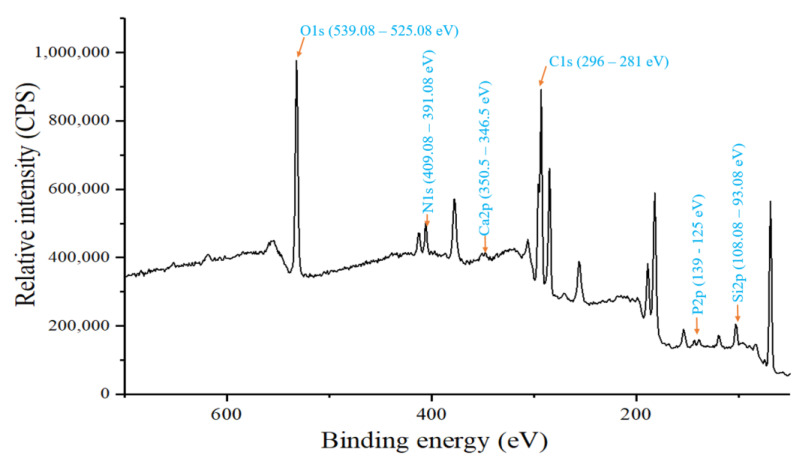
XPS survey of garden soil sample.

The availability of elements such as P, O, N, Ca, Si, and C was analyzed using XPS, as shown in Figure 1. The XPS analysis of garden soil showed the presence of oxygen at 539.08–525.08 eV, silica at 108.08–93.08 eV, phosphorus at 139–125 eV, carbon at 296–281 eV, calcium at 350.5–346.5 eV, and nitrogen at 409.08–391.08 eV. XPS analysis revealed that major peaks were observed for carbon and oxygen (Figure 1). 

### 3.2. Cr (VI) Removal by Using Soil-Derived Microbial Consortia

**Figure 2:**
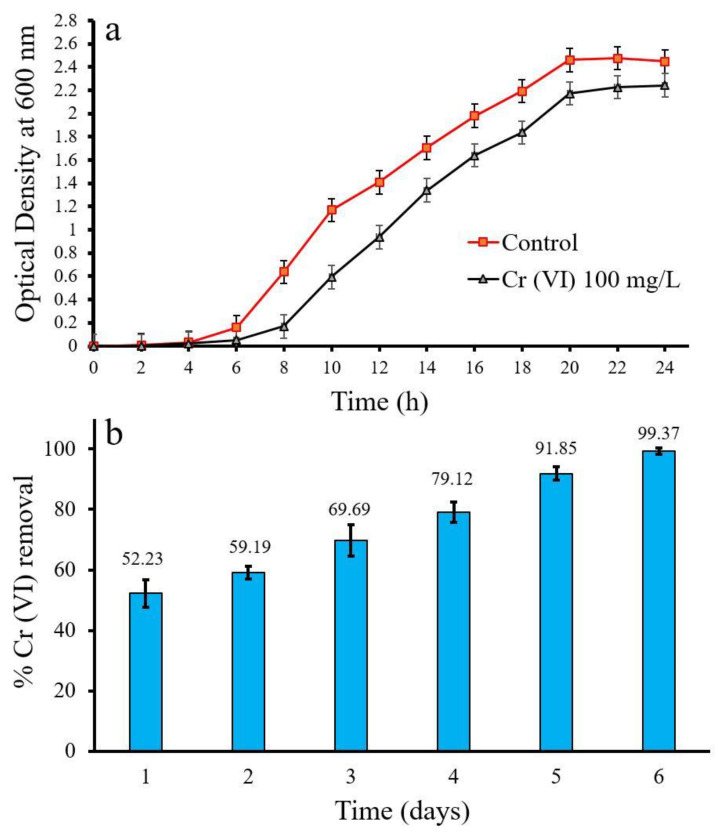
Effect of Cr (VI) on bacterial growth (**a**) and Cr (VI) removal using bacterial microbial consortia (**b**).

The effects of Cr (VI) on bacterial growth and Cr (VI) removal efficiency are shown in Figure 2.

The bacterial cells were well grown in the control compared to Cr (VI)-containing media (Figure 2a). The bacterial cell showed delayed growth in the 100 mg/L Cr (VI)-containing media. The growth inhibition of bacterial cells in the heavy-metal-containing media confirmed the Cr (VI)’s toxicity to the bacterial cells. 

The removal of Cr (VI) from the aqueous medium by bacterial mixed culture was observed, as shown in Figure 2b. The maximum removal of Cr (VI) was achieved as 99.37% at 100 mg/L Cr (VI) after 6 days of incubation. The minimum Cr (VI) removal (52.23%) on the first day and Cr (VI) removal was enhanced with incubation time, and maximum removal was reported on the sixth day. 

### 3.3. High-Throughput Data Analysis

**Figure 3:**
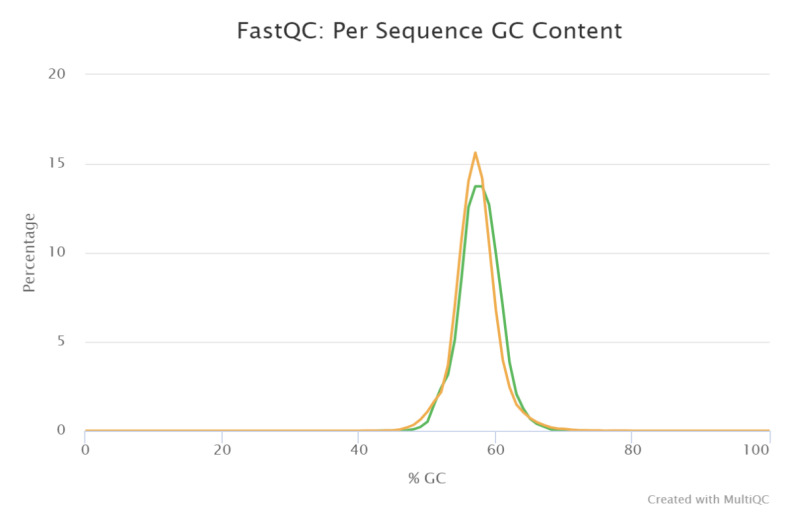
Using FastQC tools, we show the GC Content of the garden soil sample. Yellow line indicates to the theoretical distribution and green line indicates to the GC counts per read.

In the present study, the Illumina MiSeq sequencing platform was used for paired-end sequencing of DNA fragments. FastQC software revealed that metagenomic reads containing 56.5% GC content are shown in (Figure 3).

After prefiltering the datasets, metagenomic analysis of the garden soil sample shows the abundance of bacterial and archaeal microbial communities.

### 3.4. Taxonomic Composition Analysis

**Figure 4:**
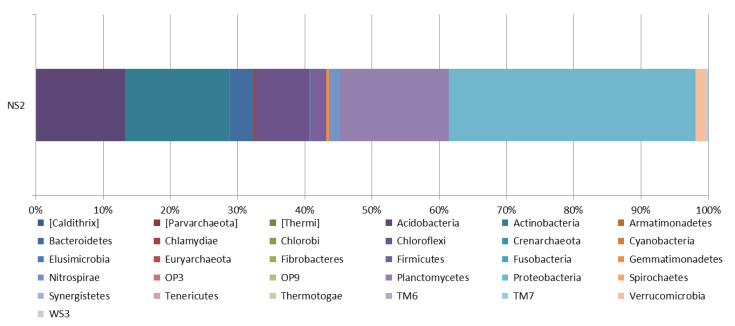
Relative abundance of all bacterial and archaeal phylum in the garden soil sample.

The metagenome sequence data were submitted to NCBI SRA under accession number SRR15186789 and BioProject number PRJNA747916. The taxonomy classification results consist of prokaryotes such as bacteria and archaea in the garden soil sample (Figure 4). 

Figure 4 represents garden soil samples, mainly including Proteobacteria, Planctomycetes, Actinobacteria, Acidobacteria, Chloroflexi, Bacteroidetes, Firmicutes, Gemmatimonadetes, Nitrospirae, Verrucomicrobia, TM7, WS3, Chlamydiae, Elusimicrobia, Cyanobacteria, Chlorobi, OP3, Armatimonadetes, TM6, Spirochaetes, Tenericutes, Fusobacteria, Synergistetes, OP9, Thermotogae, Caldithrix, Thermi, and Euryarchaeota. Parvarchaeota and Crenarchaeota are also detected. 

Proteobacteria was most abundant in the garden soil sample, followed by *Planctomycetes*, *Actinobacteria*, *Acidobacteria*, *Chloroflexi*, *Bacteroidetes*, and *Firmicutes*; the abundance of these bacteria indicated that these species play an essential role in the soil sample. 

**Figure 5:**
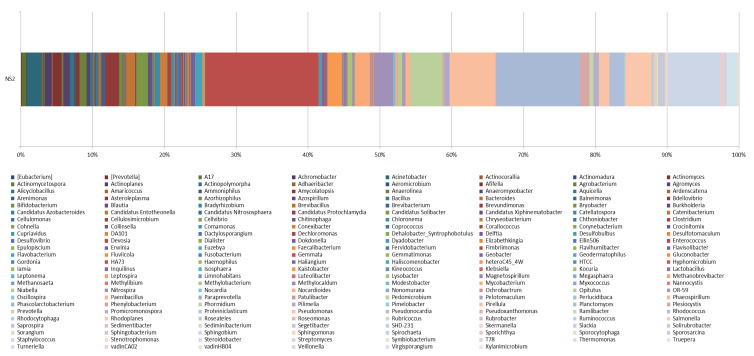
Show the microorganism at the genus level in garden soil.

In this study, Gemmata, Planctomyces, Steroidobacter, Pirellula, Pedomicrobium, Rhodoplanes, Nitrospira, Mycobacterium, Pseudonocardia, Acinetobacter, Bdellovibrio, Kaistobacter, Candidatus Solibacter, Virgisporangium, Candidatus Entotheonella, Plesiocystis, Afifella, Anaerolinea, Virgisporangium, Agromyces, Actinoplanes, Hyphomicrobiium, Devosia, Bradyrhizobium, Agrobacterium, and Pseudomonas were present in garden soil (Figure 5). 

**Figure 6:**
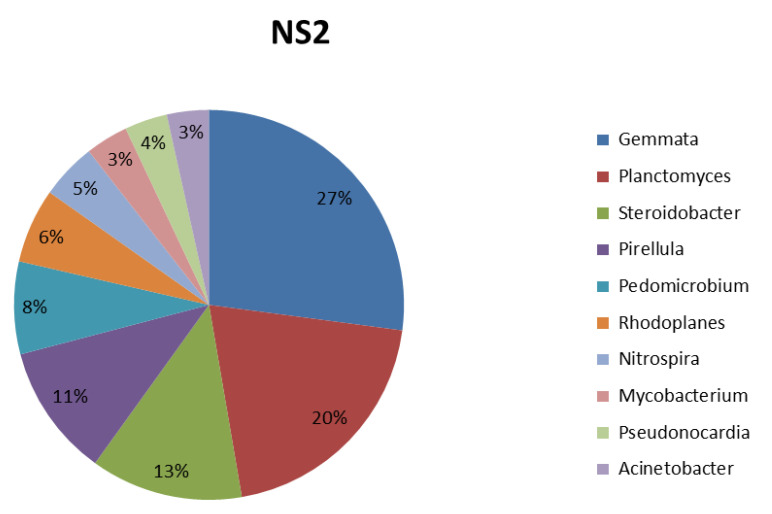
Top 10 abundant genera in garden soil samples.

In this study of soil samples, the most dominant genera—Gemmata, Planctomyces, Steroidobacter, Pirellula, Pedomicrobium, Rhodoplanes, Nitrospira, Mycobacterium, Pseudonocardia, and Acinetobacter—are shown in Figure 6.

Some of these genera were reported in the investigation of Walters et al. [45] on maize rhizobium. The highly enriched microbial community at the genus level shows their environmental characteristics, as depicted in Table 1.

The maximum abundant genus in garden soil shows their taxonomy and OTU number in Table 2.

## 4. Discussion

The XPS elemental analysis of soil confirmed the presence of O, C, Si, P, Ca, and N. Lopez-Nunez et al. [54] investigated elemental analysis in the soil and reported the presence of silica, calcium, and phosphorus in the soil sample. Zocche et al. [55] analyzed the heavy-metal composition in vegetable farming soil and reported silica, phosphorus, calcium, and zinc availability of the soil. Krupenikov et al. [56] reported elemental analysis in the clay soil and reported calcium and silica as major elements. 

The growth of mixed bacterial culture was inhibited as the Cr (VI) concentration in the growth medium increased. Upadhyay et al. [57] isolated *Bacillus* sp. MNU16 from coal-mining water and reported that the bacterial isolate could be grown at high concentrations of Cr (VI). Masood and Malik [58] observed that *Bacillus* sp. FM1 growth was inhibited in the Cr (VI) containing medium compared to the control. Cr (VI) is attached to the bacterial surface in the growth medium and enters the cell via numerous cell-surface receptors [59]. Cr (VI) accumulates in the intracellular space of bacterial cells. The intracellular Cr (VI) is reduced into Cr (III), which is involved in the cell metabolic pathways and binds to the heavy binding protein [29].

These bacteria provide primary functions relevant to the biogeochemical cycle. Proteobacteria play critical roles in the nitrogen cycle, including nitrifying bacteria (*Nitrospira*) [60]. Iliev et al. [61] reported in their study that Proteobacteria were dominant in sediments or soils. Proteobacteria play an important role in the metabolic processes related to global carbon, nitrogen, and sulfur cycling in natural and artificial wetlands. The outcome of this study is similar to that of the study by Iliev et al. [61] on wetland soil. According to Gupta et al. [17], Proteobacteria were more prevalent in garden soil (61.74%) than in hospital soil (47.28%). Among Proteobacteria, *Rhizobiales* were enriched in the garden soil samples. This group was recognized to perform an essential role in nitrogen fixation. This can correlate with increased nitrogen content in garden soil. According to some researchers, garden soil has the highest microbial diversity of any soil [17]. Planctomycetes are the second most common bacteria in our samples. Actinobacteria are the third most common bacteria and play an important role in soil breakdown, humus construction, and nitrogen fixation in the soil system. Actinobacteria members are also economically and agriculturally relevant as a source of antibiotics and pesticides [62,63]. Chloroflexi is a photosynthetic bacteria present in garden soil samples. 

Environment-related taxa such as chemoautotrophs characterized in our study are involved in ammonium oxidation such as *Nitrospira* and *Bacillus*. Iron and manganese oxidation such as *Pedomicrobium* and *Geobacter* were identified in our study. These bacterial genera are also involved in N_2_ fixation [64]. Szymańska et al. [65] reported that *Pedomicrobium*, more frequent in sugar beet, represents *Rhizobiales*, an order known for organisms that establish beneficial interactions with plants which encompasses plentiful bacteria with nitrogen-fixing competence. 

In our study, *Gemmata* was dominant in garden soil. Garg et al. [66] reported that the genus *Gemmata* was absent in imidacloprid-applied soil. Garg et al. [66] also reported that the soil microbial community of garden soil had higher microbial diversity, as reported. The garden soil microbial diversity is associated with plant resistance to pathogens [66].

*Agromyces* and *Bradyrhizobium* bacterial genera were also found in our study, which confirms the superior fertility of the garden soil sample. Wang et al. [67] reported that *Bacillus*, *Agromyces, Lysobacte, Pseudonocardia*, and *Bradyrhizobium* were found in the majority in the healthy soil sample. Authors also investigated that these bacteria improve soil nutrients, encouraging plant growth, and monitoring soil-borne diseases. *Rhodoplanes* bacteria were one another dominant bacterial genera found in the garden soil sample in our study. These bacteria are phototrophic bacteria present in the rhizosphere soil [49]. Srinivas et al. [49] reported that *Rhodoplanes* were isolated from the rhizosphere soil of paddy. Oliveira et al. [64] reported that *Nitrospira* is found in vegetated soil, including unfertilized grassland soil. These microorganisms are found in the area surrounded by vegetative plants and trees. Li et al. [50] investigated that *Nitrospira* is an abundant bacterium that plays an essential role in the nitrification of fertilized soils. *Nitrospira* is found in diverse environments and plays an important role in the nitrogen cycle [68,69]. Hruska and Kaevska [70] reported that mycobacterium is non-tuberculous in mycobacteria detected in soil. The soil was easily contaminated by fertilization with manure or liquid dung, or water contaminated by animal faeces. 

Genera *Mycobacterium* and *Pseudonocardia* were found in our study within the Actinobacteria group. *Mycobacterium* are generally free-living saprophytes and are the causative agents of a broad spectrum of human diseases. *Pseudonocardia* is a healthy plant-associated bacteria that promotes plant growth [48]. *Mycobacterium* was significantly enriched in the beech rhizosphere in the two most acidic and nutrient-poor soils [51]. Sit et al. [52] reported that *Pseudonocardia* isolated from the soil are considered rare actinomycetes. Holmes et al. [48] investigated *Pseudonocardia* species associated with *Acromyrmex* ants and found evidence to support the concept that *Pseudonocardia* might be a potential source of novel antimicrobials. Because they aggressively cut new leaves and eat them, *Acromyrmex* are sometimes known as “leafcutter ants”.

The Genus *Acinetobacter* found in our study plays an important role in the degradation of various long-chain dicarboxylic acids and aromatic and hydroxylated aromatic compounds. Iliev et al. [61] reported that the genus *Acinetobacter* could transform nitrogen through heterotrophic nitrification and aerobic denitrification. Some bacterial species within Firmicutes are present in fewer amounts in the garden soil sample. Soil bacteria are essential for the decomposition of organic matter from plant products [71]. Nitrogen-fixing microbes are present in the soil and some plants, such as peas and beans. Without microbes, the carbon and nitrogen cycles would not exist [72,73].

## 5. Conclusions

This study evaluated the distribution of bacterial diversity in garden soil and the role of the soil-derived bacterial consortia in mitigating toxic Cr (VI) ions from wastewater. The most abundant genera in garden soils are *Gemmata*, *Planctomyces*, *Steroidobacter*, *Pirellula*, *Pedomicrobium*, *Rhodoplanes*, *Nitrospira Mycobacterium*, *Pseudonocardia*, and *Acinetobacter*. The garden soil bacteria were also associated with improving soil fertility and promoting plant growth. The elemental analysis of garden soil indicated that carbon and oxygen were abundant in the soil. Additionally, nitrogen, silica, phosphorous, and calcium were also present in the soil. The soil-derived bacterial consortia removed 99.37% Cr (VI) from the water. This study revealed that the soil microbial community could help promote plant growth, biodegradation of organic matter, and the removal of toxic Cr (VI) ions from wastewater.

## Figures and Tables

**Table 1:** Major microbial communities play an essential role in garden soil.

Bacteria (Genus)	Function (Characteristics)	References
*Gemmata*	Chemoheterotrophic aerobes	[46]
*Planctomyces*	It is found in microbial fuel cell systems; it plays a role in bioconversion and energy-transfer processes.	[46]
*Steroidobacter*	Agar-degrading bacteria,	[47]
*Pirellula*	Chemoheterotrophic aerobes play a role in the degradation of sulfated glycopolymers.	[46]
*Pedomicrobium*	More dominant in the crop field rhizosphere. Primarily found in sugar beet. It shows beneficial interaction with plants and comprises numerous bacteria with N2-fixing capability.	[48]
*Rhodoplanes*	Phototrophic bacteria are present in the rhizosphere soil of paddy.	[49]
*Nitrospira*	They are ubiquitous bacteria that play a role in the nitrification of fertilized soil.	[50]
*Mycobacterium*	It is significantly enriched in the rhizosphere soil.	[51]
*Pseudonocardia*	It is a plant-associated microbial community. It improves soil nutrients, promotes plant growth, and controls soil-borne disease. It also plays a vital role in the degradation of xylan through the production of xylanase.	[52]
*Acinetobacter*	It implies active participation in the nutrient cycle in the ecosystem. It involves the degradation of various long-chain dicarboxylic acids and aromatic and hydroxylated aromatic compounds associated with plant degradation products.	[53]

**Table 2:** Most abundant microbial diversity of garden soil sample.

OTU Number	Kingdom	Phylum	Class	Order	Family	Genus
910	Bacteria	Planctomycetes	Planctomycetia	Gemmatales	Gemmataceae	*Gemmata*
679	Bacteria	Planctomycetes	Planctomycetia	Planctomycetales	Planctomycetaceae	*Planctomyces*
424	Bacteria	Proteobacteria	Gammaproteobacteria	Xanthomonadales	Sinobacteraceae	*Steroidobacter*
	Bacteria	Planctomycetes	Planctomycetia	Pirellulales	Pirellulaceae	*Pirellula*
257	Bacteria	Proteobacteria	Alphaproteobacteria	Rhizobiales	Hyphomicrobiaceae	*Pedomicrobium*
208	Bacteria	Proteobacteria	Alphaproteobacteria	Rhizobiales	Hyphomicrobiaceae	*Rhodoplanes*
156	Bacteria	Nitrospirae	Nitrospira	Nitrospirales	Nitrospiraceae	*Nitrospira*
119	Bacteria	Actinobacteria	Actinobacteria	Actinomycetales	Mycobacteriaceae	*Mycobacterium*
119	Bacteria	Actinobacteria	Actinobacteria	Actinomycetales	Pseudonocardiaceae	*Pseudonocardia*
115	Bacteria	Proteobacteria	Gammaproteobacteria	Pseudomonadales	Moraxellaceae	*Acinetobacter*

## Data Availability

Data are contained within the manuscript.
